# BECOMING INCLUSIVE: A NECESSARY PURSUIT FOR SUPPORTING FAMILY CAREGIVERS IN HOSPITAL CARE

**DOI:** 10.1093/geroni/igad104.1883

**Published:** 2023-12-21

**Authors:** Chloe Muntefering

**Affiliations:** University of Wisconsin- Madison, Madison, Wisconsin, United States

## Abstract

Given advances in medicine and technology, shorter hospital stays, and a shrinking health care workforce, an increasing number of older adults are relying on family caregivers for support during and after hospital care. Yet, our healthcare system is often unresponsive to the needs of family caregivers. Recent consensus recommendations including the National Strategy on Family Support encourages health systems to include family caregivers in care processes of older adults. This presentation will report interview and community advisory board findings from a qualitative descriptive study of N=24 stakeholders consisting of hospitalized older adults (n=4), family caregivers (n=6), healthcare providers (n=9), and health policymakers (n=5). The goals of the study were to identify and describe existing barriers and facilitators to the inclusion of family caregivers in older adult’s hospital care. Of the participants, 87% were female and from the Midwest region, and all were Caucasian. The average age was 56 years. The resulting themes from this exploration demonstrated that inclusion of family caregivers in hospital care is influenced by personal factors of both the caregiver and the provider (caregiver availability, health literacy, education, values/priorities), interpersonal skills between these two parties (communication style and methods), hospital environment/resources (accessibility, comfortability, staffing, documentation), and social climate/policy (Covid-19 Restrictions, HIPAA, Power of Attorney). Findings from this study can be used to develop or adapt healthcare system policies and practices, fulfilling Outcome 2.1 of the National Strategy on Family Support’s recommendation for recognizing family caregivers as essential members of the care team.

